# In a maternity shared-care environment, what do we know about the paper hand-held and electronic health record: *a systematic literature review*

**DOI:** 10.1186/1471-2393-14-52

**Published:** 2014-01-30

**Authors:** Glenda Hawley, Tina Janamian, Claire Jackson, Shelley A Wilkinson

**Affiliations:** 1 APHCRI Centre of Research Excellence in Primary Health Care Microsystems, School of Medicine, Discipline of General Practice, University of Queensland, Herston 4029, Brisbane, Australia; 2 Mater Research, Mothers & Babies Theme, Mater Health Services, South Brisbane 4101, Australia; 3 Department of Nutrition and Dietetics, Mater Health Services, South Brisbane 4101, Australia

**Keywords:** Paper hand-held record, Electronic health record, General practitioner, Maternity, Antenatal, Shared-care, Hospital clinician

## Abstract

**Background:**

The paper hand-held record (PHR) has been widely used as a tool to facilitate communication between health care providers and a pregnant woman. Since its inception in the 1950s, it has been described as a successful initiative, evolving to meet the needs of communities and their providers. Increasingly, the electronic health record (EHR) has dominated the healthcare arena and the maternity general practice shared-care arrangement seems to have adopted this initiative. A systematic review was conducted to determine perspectives of the PHR and the EHR with regards to data completeness; experiences of users and integration of care between women and health care providers.

**Method:**

A literature search was conducted that included papers from 1985 to 2012. Studies were chosen if they fulfilled the inclusion criteria, reporting on: data completeness; experiences of users and integration of care between women and health care providers. Papers were extracted by one reviewer in consultation with two reviewers with expertise in maternity e-health and independently assessed for quality.

**Results:**

A total of 43 papers were identified for the review, from an initial 6,816 potentially relevant publications. No papers were found that reported on data completeness in a maternity PHR or a maternity EHR, in a shared-care setting. Women described the PHR as important to their antenatal care and had a generally positive perception of using an EHR. Hospital clinicians reported generally positive experiences using a PHR, while both positive and negative impressions were found using an EHR. The few papers describing the use of the PHR and EHR by community clinicians were also divergent and inconclusive with regards to their experiences. In a general practice shared-care model, the PHR is a valuable tool for integration between the woman and the health care provider. While the EHR is an ideal initiative in the maternity setting, facilitating referrals and communication, there are issues of fragmentation and continued paper use.

**Conclusions:**

There was a surprising gap in knowledge surrounding data completeness on maternity PHRs or EHRs. There is also a paucity of available impressions from community clinicians using both forms of the records.

## Background

The paper hand-held record (PHR) has been a successful and integral tool used in maternity shared-care for many years. Hamilton introduced the ‘Co-op (co-operation) card' in 1956 in the United Kingdom (UK) and since this time, women and clinicians have used some version of the PHR to record maternity care [1]. The PHR continues to be widely used in the UK and also in Australia and New Zealand (NZ) [2]. The woman carries the PHR with her and the care given is documented at each visit to either the community clinician or the hospital. Evidence shows that PHRs improve communication between health care providers, reduce anxiety and increase women's involvement in their care [3]. The benefits of the PHR have been demonstrated in previous, mainly descriptive studies but little formal evaluation has been done on the data collected or on the experiences of health care providers using the PHR.

Increasingly, the use of a patient electronic health record (EHR) has become evident. Internationally much work has been done on evaluating the implementation of EHRs in a variety of health settings. Implementation issues of standardising processes, safety and security, promoting evidence based practice, ease of use, easing workload and using less paper charts have all been cited [4]. The EHR is designed to use information in a digital format that can be used by both patients and health care providers, from anywhere, at any time [4]. Digital records are accessed using a variety of devices and media, including: USB (portable memory) stick and web-enabled interfaces of personal computers, smart phones or tablets.

The EHR was introduced in Australia in the 2010/2011 federal budget and the Australian Government Department of Health and Aging (DOHA) with the National e-health Transitory Authority (NEHTA) announced an investment over 2 years to deliver a national Personally Controlled EHR (PCEHR) [5]. The EHR is proposed to greatly enhance both the quality and the timeliness of available healthcare information. It is suggested that it will allow consumers to have access to information, better manage their health care online and be beneficial to health care providers through improved sharing of clinical information.

Access to best practice maternity care is a major priority on the Australian national health agenda. To address the fragmentation of care currently provided (in alignment with the PCEHR), a maternity EHR has been developed and is currently being trialed in a general practice (GP) shared-care setting. Shared-care is seen as a service provided between the primary and secondary care sectors, with GPs as the fundamental central component [6]. The EHR in a maternity shared-care setting aims to integrate clinical care between GPs, midwives, allied health professionals and the woman herself. Integration between these care sectors is the significant factor required for effective and safe management of pregnant women, with many approaches being identified [6]. There is evidence of using the PHR as an integration tool between health professionals, but determining if the PHR or the EHR better facilitates this integration is not known. This review was undertaken to investigate the differences in using a PHR and an EHR in a GP maternity shared-care environment with regards to data completeness, experiences of users and integration of care between women and health care providers.

## Methods

### Search strategy

A search of Medline, OVID, CINAHL, and Embase was conducted, incorporating key words, subject headings and MeSH terms. Papers were excluded if not written in English. To capture all relevant information on the introduction of the maternity record, all evidence levels were included and initially no date restrictions were applied. Once results were first perused, it was decided to only include papers dated after 1985 to capture most of the literature surrounding PHR's. Only full text papers were included. The search was conducted in three stages. The first stage was an open search investigating the maternity health record in paper and electronic formats. Additional topics of experiences and perceptions using ease of functionality and barriers to use was added to the search. The second stage was conducted to extract papers examining data completeness in health records and the third stage focused on the integration of maternity shared-care model health services.

**Figure 1:**
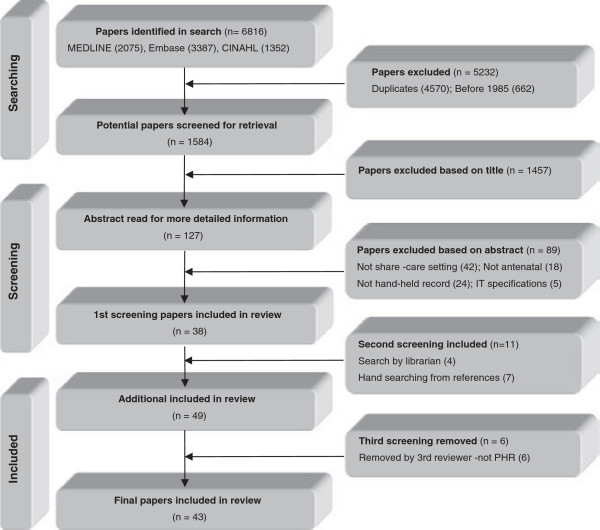
Study selection flow diagram.

Initial search strategy terms included variations of: matern*, pregnan*, antenat*, prenat*, perinat*, midwi* AND record*, chart*, note*. Using keywords and MeSH subject headings, the results were too broad. The search was narrowed down by using focused MeSH for the medical records terms. Truncation for the words perinat*, card*, chart*, note* were then removed. As result numbers continued to be large, the search was narrowed by using the adjacency operator (adj3), with key words only. The second search built on the first by including MeSH term variations of “data” AND “quality”, “completeness” and “accuracy”. The third search was conducted with MeSH terms including "family physician", general practitioner", "integrated", interdisciplinary", "perinatal care", "computerised patient record", patient access to records", "medical records, personal", to find papers specific to maternity integration in GP shared-care. The PRISMA based flow diagram is demonstrated in Figure 1[7]. The search was verified by two librarians experienced in systematic reviews (see Additional file 1).

### Study selection

Table 1 summarises the inclusion and exclusion criteria of the published papers in the review. Quantitative and qualitative papers were included if they contained information relevant to three key elements:

1. Data completeness in a PHR and EHR,

2. Experiences of women and health providers when using a PHR and EHR for perceptions, satisfaction and usability, and

3. Maternity shared-care as an integrative model using a PHR and EHR for teamwork, clinical input and process deliverables.

**Table 1:** Inclusion and exclusion criteria for the systematic review

**Review concepts**	**Inclusion criteria**	**Exclusion criteria**
EHR	1. An EHR is defined as a system that operates between hospital, community setting and patient The record is accessible by hospital clinician, patient (or woman) and community clinician.	1. Any electronic system that operates within a hospital (including linking hospital departments) and is not accessible by external facilities.
PHR	1. Person can include “patient”, “client”, “woman”	1. Any paper record that is an in hospital based medical chart or notes.
	2. Paper record is portable and hand-held.	
	3. Record can be known “notes”, “chart”, “card”. Shared-care record can be known as “home-based record” in developing countries.	
Shared-care environment	1. Setting that is defined as a joint partnership between a specialist (or secondary) and a primary care setting.	1. Secondary setting where there are attached satellite units of the main facility.
	2. Care provided is for particular patient (or woman).	
	3. Can be in a developed or developing countries. In developing countries, secondary setting may be defined as a “clinic” or “centre”	
Community General Practitioner (GP)	1. May be defined as a community “physician”, “practitioner”, “provider” May also work in secondary setting.	1. Private obstetrician

### Screening and data extraction

All search strategy results were entered into EndNote X6® (Thomson Reuters, New York, NY, USA) and screened by all titles and abstracts, by one author. An independent search of the literature was conducted by an experienced librarian to verify selection of papers. To gain a global perspective, both international and Australian published papers were included in the review.

A data extraction grid was developed by two authors, through previous research related to primary health care and shared-care settings. Characteristics of included papers were summarised by type of study, country, year, methods, setting and population. Findings from the papers were collated and reviewed for inconsistencies by two reviewers. Non-relevant findings were removed and additional information was included as necessary.

Data findings were identified and grouped as comparisons on the use of different versions of the record (PHR or EHR). Outcome data collected was included as available. Descriptive findings were classified according to the women, hospital and community clinicians, and compared for synthesis or combinations of findings.

### Study quality assessment

Study quality was appraised using a mixed methods research scoring system developed by Kmet, which proposes assessment criteria for evaluating primary research papers from a variety of fields (see additional file 2)*.* All included papers were screened by one reviewer and checked for reliability by a second reviewer. If a paper utilised a mixed-method approach, then both quantitative and qualitative assessments were conducted. Thirty papers were assessed qualitatively. Seventeen were quantitative and four were assessed using both quantitative and qualitative assessment criteria. Despite six papers scoring a low rating (including editorials, responses, communications and one abstract), they were included as supplementary papers, providing contextual information from unique settings [8-13].

### Data synthesis

Papers included have been examined by considering the following three elements, each with separate components.

#### Data completeness

The papers were required to report data on key evidence based antenatal variables, or as obstetrically important before guidelines were available. Data completeness could be presented as frequencies and could be stand-alone or comparative data.

#### Experiences of users

As the interpretation of 'experience' can be broad, the term was defined and explained by adding the words: perceptions (feelings), satisfaction (likes and dislikes) and usability (functionality, access). Papers were included if the described perspectives of experience were clearly documented from the users of the maternity record, including the pregnant woman herself, hospital clinicians (midwives, allied health doctors) and community practitioners (including GPs participating in a shared-care program).

#### Integration of care

Papers that described the components of integration in a maternity shared-care model with community clinicians (including GPs) were included. The papers need to have mentioned integration in terms of teamwork (collaboration modalities), clinical input (results, visit data) and process deliverables (how to do things, reporting, guidelines and communication strategies). The papers described the integration using or proposing to use either a PHR or an EHR.

### Characteristics of included papers

Included papers are summarised in Table 2, by the review concepts identified in each of them. Most of the papers were published from the United Kingdom (n = 17) and Australia (n = 16). The remaining papers originated from: USA (n = 2); Zimbabwe (n = 1); Switzerland (n = 2); Denmark (n = 1); Malta (n = 1); Finland (n = 2); Canada (n = 1). There were 37 original papers. Nine papers used comparison data and 28 presented descriptive findings. Three papers were reviews, two were responses to original papers and one was a Cochrane review.

**Table 2:** Summary of papers included in the systematic review

	**Study** Author (ref) (Country)	**Question 1. Data completeness**		**Question 2.Experiences**						**Question 3.Integration**	
		- Only antenatal variables identified		- Perceptions						- Teamwork	
		- Data included as present or not present		- Satisfaction						- Clinical input	
				- Usability						- Process deliverables	
				- Access							
				**Women**		**Hospital Clinicians**		**Community Clinicians (GPs)**			
		**PHR**	**EHR**	**PHR**	**EHR**	**PHR**	**EHR**	**PHR**	**EHR**	**PHR**	**EHR**
**1**	Elbourne [14] (UK)			X		X					
**2**	Lovell [15] (UK)			X		X					
**3**	Homer [2] (AUS)			X							
**4**	Brown [16] (UK)			X							
**5**	Wilkinson [17] (AUS)			X		X					
**6**	Webster [18] (AUS)			X							
**7**	Phipps [3] (AUS)			X							
**8**	Toohill [19] (AUS)					X					
**9**	Kiran [8] (UK)			X							
**10**	Holmes [20] (UK)			X		X					
**11**	Draper [9] (UK)			X		X					
**12**	Shah [21] (Switzerland)			X							
**13**	Mahomed [22] (Zimbabwe)			X		X					
**14**	Turner [23] (2011)			X		X					
**15**	Patterson [24] (AUS)			X		X				X	
**16**	Wood [25] (UK)			X						X	
**17**	Thomas [26] (UK)			X		X		X		X	
**18**	Halloran [27] (AUS)							X		X	
**19**	Wackerle [28] (Switzerland)				X						
**20**	Fawdry [29] (UK)						X				
**21**	Curly [10] (UK)										
**22**	Homer [30] (AUS)						X				
**23**	Winthereik [31] (Denmark)				X		X		X		
**24**	Jones 2002 [32] (UK)						X				
**25**	Jones 2004 [33] (UK)						X				
**26**	Henwood [33] (UK)						X		X		
**27**	Hart [34] (UK)						X				
**28**	Shaw [35] (Canada)				X						
**29**	Kouri [36] (Finland)						X				
**30**	Tindale [37] (UK)						X				
**31**	Lombardo [38] (Aus)									X	
**32**	Gunn [11] (Aus)									X	
**33**	Sosa [12] (Aus)									X	
**34**	Nel [39] (Aus)									X	
**35**	Field [40] (UK)									X	
**36**	Haertsch [41] (Aus)									X	
**37**	Bedford [42] (UK)									X	X
**38**	Jackson [43] (AUS)									X	
**39**	Dawson [44] (AUS)									X	
**40**	Angood [45] (USA)										X
**41**	Hakkinen [46] (Finland)										X
**42**	Savona-Ventura [47] (Malta)										X
**43**	Knowlden [13] (AUS)										X

## Results

### Data completeness in a maternity record

There were no papers found in the literature reporting specifically on data completeness in a maternity PHR or a maternity EHR, in a shared-care setting.

### Women's experiences using a PHR or EHR in a maternity setting

Table 3 provides a summary of women’s experiences in using PHR or EHR in a maternity setting. Specific details relating to perceptions and satisfaction and usability and access are outlined below.

**Table 3:** Summary of Women's experiences using PHRs and EHRs in a maternity setting

**Experience**	**PHR maternity record**	**EHR maternity record**
*Perception*	• Having more ownership and feeling more in control of pregnancy	• Positive impressions
	• More confidence, responsibility	• 80% with record on USB felt safer and would use again
	• Perceived as getting better care	• Few concerns over confidentiality
*Satisfaction*	• High level of satisfaction, less anxious	• High level of satisfaction using an internet device
	• Communication improved	
*Usability*	• Easy to use	• Electronic notes useful, easy to understand
	• Prefer to carry own notes and would do again	• Assisted with education, remembering appointments
	• Improved availability to education	
	• Some findings of writing hard to read and difficult to carry	
*Access*	• Generally did not lose record	• Improved partner involvement
	• Good access to information for partner, family and friends	• Some issues with not being able to access record
		• When data missing from record, expected to recall information

#### Perceptions and satisfaction

A common perception identified was that women reported to have greater ownership and feeling more in control of their pregnancy when using a PHR [2,3,8,14,15,18,23]. It was noted that carrying notes gave women more confidence and women felt more responsible, involved and in charge of their health [3,9,22]. Women were documented as thinking the PHR was a good idea, important and perceived themselves to be getting better care when they had more information [2,3,21,22]. However, one study reported a perception of one third of women who used an antenatal record card felt it had little impact on their care [24]. A generally high level of satisfaction was reported by women when they carried their PHR [2,3,9,14-16,18,20,21,23],[25]. Papers reported women thought that talking to midwives and doctors was easier and communication was improved when carrying their own full PHR [2,14-16,20,25,26]. Two papers reported on women being less anxious when using a full PHR [2,14].

Two papers reported on positive impressions of the EHR [28,35]. Wackerle found that four fifths of women who had their maternity notes on a USB stick felt safer and Shaw indicated that women felt a high level of satisfaction when using an internet device [28,35]. Although women expressed a few concerns over confidentiality, women using the USB stick said they were satisfied with the pregnancy care and would repeat their experience [28].

#### Usability and access

Women were reported as thinking that the PHR was useful and easy to use [8,20,21]. Most papers reported that women looked after their notes, would prefer to carry their own paper notes and would do so in the next pregnancy [2,14,15], [2,8,9,16,22]. Carrying the full PHR was also noted to improve opportunities to receive reminders and educational information and also motivated them to learn more about pregnancy [3,21-23]. Thomas documented that over fifty percent of women would prefer to have shared-care with the GP, midwife and obstetrician [26]. However, some papers suggested that different versions of the full PHRs were difficult to use and carry, harder to read and that documentation and efficiency was not improved [2,9,14,17,20]. Despite concerns of decreased access and that women would lose their PHR, few women did not bring their PHR to appointments [2,3,14,15,20,23]. Papers also documented that women found it advantageous that their husband, family and/or friends could view their record [2,9,15,22]. One study reported that about half the women presented with their PHRs, despite issues with women being mobile and travelling long distances to receive maternity care [22]. Phipps noted that women described the PHR as a tangible and important link to the pregnancy fostering sharing of information, while another study found that women did not want access to difficult or problematic information [3,9].

Women reported that EHR notes were considered useful [28,35]. Shaw noted that women thought the EHR was easy to understand and assisted in educating, making decisions and remembering appointments [35]. Wackerle reported that two thirds of women regularly used the USB record, a quarter used the USB record after every consultation and less than one tenth shared the USB record with a community doctor [28]. This paper also suggested improved partner involvement using the USB record [28]. Two papers did suggest that women could not or did not access their EHR [28,31]. Winthereik provided valuable insight into women being responsible participants in their own health care. When using an EHR, if data was missing or the record was not available, the women were expected to recall information that had been communicated or documented on their record [31].

### Hospital clinicians' experiences using a PHR or EHR in a maternity setting

Table 4 provides a summary of hospital clinician's experiences in using PHR or EHR in a maternity setting. Specific details relating to perceptions and satisfaction and usability and access are outlined below.

**Table 4:** Summary of Hospital Clinicians’ experiences using PHRs and EHRs in a maternity setting

**Experience**	**PHR maternity record**	**EHR maternity record**
*Perceptions*		• Both positive and negative perceptions General acceptance, although midwives showed disinterest, confusion and not integral to their role
*Satisfaction*	• Satisfied with using record	• Increased reliability of information
	• Generally improved communication	• Improved legibility, less duplication Despite prediction of paperless future, paper continues to be a reality
	• Communicating with midwives sometimes problematic	
*Usability*	• Some issues with hard to read, increasing workload	• Considered time consuming – to print reports
		• Links to educational resources useful
*Access*	• Positive overall return rate at visit presentation	• Privacy, confidentiality issues
	• Some problems retrieving information if record was forgotten	• Restricted and lack of access to hospital or personal computer
	• Concern over documenting sensitive information	• Frustration when information not available – not woman’s role to recall

#### Perceptions and satisfaction

Generally, clinicians were noted as reporting satisfaction with using the PHR, stating that it improved communication with women in their care [20,23,24,26,27]. One paper did consider that communicating with midwives was at times problematic when using the PHR [26].

Five papers elucidated both positive and negative perceptions of using the EHR [29,34,36,37,48]. The positive perceptions reported were varied and included a general acceptance of: increased reliability, faster transmission of information, reduced medical errors, access anywhere, less duplication, less use of paper and improved legibility [29,30,34,36,37,48]. However, there were suggestions of problems with standardisation and non-necessity of using an EHR [10,29,33,34,36,37,48]. There was a reported lack of interest in using the EHR by midwives, who reportedly found the interface problematic and were confused about what a patient EHR was [34,36,48]. Midwives expressed disinterest and considered the EHR not integral or relevant to their role [33,48]. Two authors commented on the use of paper related to using an EHR. One author considered that in reality, paper will continue to be a necessity in areas where the user is not online, while another reported that storing images of paper may alleviate this issue of excessive paper [10,29].

#### Usability and access

Three papers referred to problems with PHR use, including a version of the PHR that had an accompanying educational component [17,24,26]. Negative issues reported about PHR use included: the record was hard to read and time consuming to use, that there were too many prompts for health professionals, that its use resulted in an increased workload, and that the PHR created more administrative load [17,24,26].

Two included papers specifically reported on PHRs being available in a positive and negative context when needed, or at presentation to hospital in the antenatal period [15,19]. One paper reported a positive finding of over two thirds return rate of the PHR at presentation in a busy antenatal assessment unit [19]. However, another paper reported findings of doctors not being able to retrieve information easily from the PHR and not having enough room to document problems or write individual comment [20]. There was also a concern about women having access to sensitive or difficult information such as a ‘problem with the baby’ when using a PHR [9]. Two papers did find that women did not necessarily have records with them or refer to their record [20,23].

Despite reports of implementing an EHR being expensive, this factor was not thought not to be prohibitive to the introduction of such a record [29,30]. Hospital clinicians considered data management (recording and retrieving data) from an EHR was time consuming, particularly for tasks of accessing histories and generating reports [32,48]. Functionalities incorporated into specific EHRs found favourable by staff included: links to educational resources, the obstetric calculator, women friendly language incorporated and a necessity to keep sensitive information confidential [30]. Factors relating to access were negatively described in terms of: issues of dealing with privacy of information, restriction or difficulty accessing and ensuring any data entry or editing could be linked to a person [30,36,37]. Some papers noted staff issues of difficulty using an EHR as, concerns over lack of access to a personal computer and also problems with linking information between hospital and community systems [30,32,36]. One paper found that midwives and doctors were frustrated when information was not available in the EHR. When this occurred, the woman was expected to recall missing information, which was not considered her responsibility [31].

### Community clinicians' experiences using a PHR or EHR in a maternity setting

Table 5 summarises the lack of information available, as reported from community clinicians. No papers were found that reported on perceptions, satisfaction, or usability with a PHR or an EHR.

**Table 5:** Summary of Community Clinicians' experiences using PHRs and EHRs in a maternity setting

**Experience**	**PHR maternity record**	**EHR maternity record**
*Access*	• Did help to educate women, pictures useful	• GPs expected to fill in blanks or missing results
	• Divergent findings of accessing PHR Accessed 51% of times during an antenatal visit	• Reluctant to share information – may be losing more than gaining

#### Access

Two papers with divergent findings about access to a PHR, were found. Holmes reported on experiences of community clinicians accessing the PHR while caring for women, finding that GPs accessed the record about half of the time, about one fifth accessed the record occasionally and over a quarter never asked about the record [20]. Conversely in a community setting in Zimbabwe, clinician's accessed the record to educate women, with information presented as pictures or figures [22].

No papers were found that reported on community clinicians' perceptions or satisfaction with an EHR. The papers that reported on GPs experiences using an EHR tended to be negative. This originated from GPs being expected to fill in laboratory results when they were missing in the EHR [31], or from issues around ownership of information. One paper described GPs as reluctant to contribute information freely to other providers. They felt their practice health records were already comprehensive [33].

### Integration of care using a PHR and an EHR in a maternity setting

#### Teamwork

Papers reporting varying views of how the GP shared-care model operates using the PHR are summarised in Table 6. One survey reported that GPs found teamwork a challenge, citing issues of communication and role distinctions with and between community and hospital clinicians. From this survey recommendations were presented, including the introduction of the PHR to improve communication between health care providers and the woman [11]. Another paper commented on the shared-care model working well, with processes already being formalised, including the PHR [12].

**Table 6:** A summary of how the use of the PHR and EHR has facilitated integration of care in a shared-care model

**Components**	**PHR maternity record**	**EHR maternity record**
*Teamwork*	• Differing views on shared-care model	• Current electronic systems are stand-alone and still use paper
	• Challenges with operating as a team member with tertiary setting	• Fragmented electronic systems result in lack of communication between care providers
	• GPs expressed inter-professional issues of role distinction, requiring more respect	• Focus of electronic record is to provide a woman centred approach
	• PHR helped in model and motivated GPs to provide good antenatal care	• Woman want improved communication (email facility) between providers and self
*Clinical input*	• Provides opportunity to ensure necessary tests are performed and documented	• Has facilitated ease, timeliness of referrals, reminders and notifications
	• Sections of pathology, ultrasound assessment, history, visit schedules important	• Some information seen as sensitive not appropriate for electronic format
	• Good to prevent duplication	
	• Record should be personalised, provision for referrals and space to write notes	
	• Midwives used non-clinical parts of record more	
*Process deliverables*	• Process to formalise framework of communication between woman and carers	• Electronic record ideal in maternity arena to integrate community, woman, obstetric unit, laboratory
	• Can be used in changing or remote settings	• Structure based on guidelines and PHR
	• PHR part of process in model of care, with continuing education and practice guidelines	• Used to link specialist services to GPs

There is little information describing the integration of maternity care using an EHR. Two papers cited the current maternity hospital EHR as inadequate, outdated and still required the entry of data from a PHR [45,46]. In a 'Blueprint for Action', an EHR was integral in improving disparities in care processes, outcomes and data collection. The EHR is recognised as an important part of health care reform and assists women to have access to high-quality care. Incorporating an email capability is seen as a way to improve communication between woman and provider, however small practices or community clinics may find it difficult to transition to using EHR capabilities [45,46].

#### Clinical input

The PHR has been noted to provide an opportunity to ensure that necessary clinical tests, such as pathology, ultrasounds and visit schedules are performed and uniformly documented [11,38,39,44,49]. Two papers suggested that documentation on the record was an important part of the process to reduce duplication of scheduled visits [25,26]. Similarly, antenatal guidelines recommend that women should carry their own records to assist in the organisational process of their care and should provide an opportunity to document personal information or concerns, referral and risk assessment information [41,42].

Three authors reported that an EHR system facilitated ease and timeliness of referral and care summaries, with inclusions of reminders and notifications of new information being a positive possibility [13,46,47]. Some data were deemed as not necessary in electronic form due to its sensitive nature, and was better relayed through telephone conversations, although the issue of where to document that information was not clear [46].

#### Process deliverables

The PHR is documented to be a key component of best-practice antenatal care, providing a single document to formalise a framework of communication of important clinical and process information between health care providers and the woman [11,12,25,27,38-40,42-44]. Even in varied and remote settings, the PHR is documented to be useful in improving outcomes and promoting active involvement in care [39,40]. Also important in this model of care using a PHR was continuing education, practice guidelines, clinical rotations in antenatal clinic settings, review and accreditation [12,38,39,43].

The maternity arena is cited as a setting in which to introduce a patient EHR to integrate information between the community clinic, woman, laboratory and obstetric unit. Using requirements from antenatal guidelines, the EHR can be designed with a clear structure with a single log-in [46,47]. The EHR can be designed using the data fields identified from the PHR, but it has been noted that it should also include antenatal visit and obstetric encounter forms, to link specialist services to the GP [42,45,46].

## Discussion

This systematic review provides valuable insights into shared-care in a maternity setting, using a PHR and an EHR. Despite the large number of papers initially identified, the review highlights the lack of data completeness studies regarding the use of both PHRs and EHRs in a maternity setting. Globally, there is a current trend of moving from a PHR to EHR, which is surprising without any real evaluation or awareness of how well the data are captured or shared between health care providers using either of these records. While other papers have been identified reporting on data completeness in the maternity setting, these focus on hospital perinatal datasets or charts, rather than with a PHR [50,51]. Three papers were also found that reported on completeness of data in records in other health settings [52-54]. One in the area of child health reported that data was more thoroughly completed in a PHR than a clinic chart record [52]. Two papers reported on data completeness in an EHR [53,54]. One in diabetes care regarding documentation of HbA1C readings, the other in a general medical setting, stating that elements essential for a complete clinical history were recorded poorly when using an EHR [53,54]. This paucity of work acknowledges future research is needed in health settings, including maternity shared-care.

A large amount of literature has been published regarding PHRs, largely highlighting women’s positive experiences with its use, improving communication and improving feelings of control. Some papers have also assessed clinicians' perceptions and experiences, also identifying general satisfaction with its use. However, a small number of papers identified administrative and documentation issues (relating to format of the document, its access and privacy of information). The information available from community clinicians using the PHR centres on access for educational opportunities.

With EHRs being a priority on the national health agenda, papers are emerging that document women’s and clinicians' experiences, as well as their impact on the delivery of care. These can be difficult to compare as the EHR has been defined as many things, including a web-enabled form or a stand-alone, USB based record. Overall as with the PHR, the EHR has been documented as being well received by women. Clinicians’ experiences and perceptions reflect the wider confidence with electronic databases of reliable data. However, also reflecting familiarity (or lack of it) with specific systems, some staff are wary of its usability, particularly in relation to accessing the EHR via computers and understanding how to use it. Ideally with improved internet access, the EHR is becoming a valuable data transfer and communication portal.

Although, hospital clinicians' were frustrated with EHR issues of disinterest, accessibility, paper printing and privacy concerns, both women and hospital clinicians' generally considered the EHR useful for maternity care. Few responses were found from GPs which tended to be negative around missing data.

The review reinforces the model of maternity shared-care as being complex but advantageous to providing effective health care to pregnant women. Many papers describe collaboration in a shared-care environment, with the inclusion of a general practitioner playing an integral role, in a multidisciplinary team. The common goal in shared-care is to improve outcomes for both pregnant women and their families by a team of midwives, medical colleagues, GPs, social workers, psychologists and other allied and community workers [55-60]. In recent years, improvements have been made to integrate care providers in the shared-care model by implementing guidelines and specialised training utilising a patient centred focus. This focus requires incorporating direct links in health care provider communication and shared decision making, using consultation, referral, clinical prompts, education strategies, shared-care co-ordination and using a PHR [43,55,56,61-63]. Using the PHR in a GP shared-care model has been a successful initiative in integrating care and providing opportunities to link information between health care providers and women. While EHRs are promoted as being ideal to use in the maternity arena, this is still very difficult to confidently assess as very few studies have published findings.

This review reinforces the important role a PHR has played in integrating the woman and health care providers in shared-care. Although an EHR is considered valuable in facilitating linkage between care providers and women, the literature to date has not been conclusive in determining if the record will also be important in integrating maternity care.

### Limitations of the study

A limitation of the review is the possibility of differences in categorisations of included studies. This is possible as most studies included information on more than one category reported. As such, the results presented may be classified as pertinent to a different category as well as those presented here. The authors of the studies were not contacted to confirm the categories chosen and do not think the results would be significantly different if this had been done.

The review also acknowledges the lack of randomised controlled trials available for inclusion. This highlights the need for future RCT’s in this field of health care. In some settings, part of the EHR documentation (usually with older versions of an EHR) is associated with the PHR. The authors considered this not to be a limitation, as the review incorporated a synthesis of information, rather than a direct comparison.

Only papers written in English have been included in this review. Although this may be considered a limitation, this inclusion criteria was used for papers from both developing and developed countries.

## Conclusions

The findings from this literature review demonstrate a gap in knowledge surrounding data completeness in PHRs and EHRs in a maternity setting. The review reinforces the PHR as being an important tool for women in their maternity care and provides generally positive impressions of using an EHR. Hospital clinicians' views vary using both the PHR and EHR, while community clinicians’ views are unclear due to a paucity of available information.

### Implications

The gap in knowledge surrounding data completeness in maternity records will prompt future research. The findings of experiences and integration of care will assist policy makers to develop improved models of information access and sharing between women, hospital clinicians and GPs.

## Abbreviations

PHR: Paper hand-held record; EHR: Electronic health record; GP: General practitioner.

## Competing interests

The authors declare that they have no competing interests.

## Authors’ contributions

GH conducted initial literature search. GH and TJ designed data extraction grid and, checked included papers for final papers for inclusion and exclusion criteria. CJ made final review of included papers. GH wrote initial manuscript and CJ, TJ and SW provided extensive review of final manuscript. All authors read and approved the final manuscript.

## Pre-publication history

The pre-publication history for this paper can be accessed here:

http://www.biomedcentral.com/1471-2393/14/52/prepub

## Supplementary Material

Additional file 1 Search strategy is attached OR contact copy may be attained by contacting author (glenda.hawley@uq.edu.au). Click here for file

Additional file 2 **Can be accessed by entering**http://www.ihe.ca/documents/HTA-FR13.pdf into search browser. Click here for file
