# Supplementary material for: In a maternity shared-care environment, what do we know about the paper hand-held and electronic health record: a systematic literature review
Source: BMC Pregnancy Childbirth. 2014 Jan 30;14:52. doi: 10.1186/1471-2393-14-52 (PMC3912922; doi:10.1186/1471-2393-14-52)
Supplement: Additional file 2 — Can be accessed by enteringhttp://www.ihe.ca/documents/HTA-FR13.pdf into search browser. [file 1471-2393-14-52-S2.pdf]

HTA Initiative # 13 • February 2004

STANDARD

QUALITY ASSESSMENT CRITERIA

FOR EVALUATING

PRIMARY RESEARCH PAPERS

FROM A VARIETY OF FIELDS

Prepared by:
Leanne M. Kmet, M.Sc.,
Robert C. Lee, M.Sc.
and
Linda S. Cook, Ph.D.

AHFMR
ALBERTA HERITAGE FOUNDATION
FOR MEDICAL RESEARCH

Acknowledgements

The Alberta Heritage Foundation for Medical Research is grateful to the following persons for information and comments on the draft paper. The views expressed in the final paper are those of the Foundation.

XX

HTA Initiative # 13

Standard Quality Assessment Criteria for Evaluating Primary Research Papers from a Variety of Fields

Prepared by:
Leanne M. Kmet, M.Sc.,
Robert C. Lee, M.Sc.
and
Linda S. Cook, Ph.D.

Copyright © Alberta Heritage Foundation for Medical Research, 2004

ISBN: 1-896956-69-XX (Print)

ISBN: 1-896956-71-XX (Online)

ISSN: 1706-7855

All rights reserved.

Design: Interpret Design Inc.

This paper has been prepared on the basis of available information of which the Foundation is aware from public literature and expert opinion and attempts to be current to the date of publication. Additional information and comments relative to the information paper are welcome and should be sent to:

Director, Health Technology Assessment
Alberta Heritage Foundation for Medical Research
Suite 1500, 10104 – 103 Avenue NW
Edmonton, Alberta, Canada T5J 4A7

Tel: 780-423-5727
Fax: 780-429-3509

www.ahfmr.ab.ca

Preface

The Alberta Heritage Foundation for Medical Research (AHFMR) health technology assessment initiative series, commenced in March 2000 with "A Framework for regional health authorities to make optimal use of health technology assessment." The purpose of this series has been to provide policy and decision-makers with the best information available on how to redesign their health care structures and processes to effectively respond to the challenge of decision-making in a turbulent health care environment.

XXX

Other papers in this series are listed on the inside front cover.
Copies of these and other reports can be found at:
http://www.ahfmr.ab.ca/frames3.html

If you have any comments or suggestions to make on this paper, I would be delighted to receive your feedback.

Don Juzwishin
Director, Health Technology Assessment
Alberta Heritage Foundation for Medical Research

ABOUT THE AUTHORS

Leanne M. Kmet, M.Sc. works for the Department of Community Health Sciences, University of Calgary.

Robert C. Lee, M.Sc. works for the Department of Community Health Sciences, University of Calgary, as well as the Calgary Health Technology Implementation Unit, Calgary Health Region, and the Institute of Health Economics, Edmonton.

Linda S. Cook, Ph.D. works for the Department of Community Health Sciences, University of Calgary, as well as the Division of Epidemiology, Prevention and Screening, Alberta Cancer Board, Calgary.

Table of Contents

Introduction 1

Methods

3

Table 1: Checklist for assessing the quality of quantitative studies 4

Table 2: Checklist for assessing the quality of qualitative studies 4

Results 6

Table 3: Inter-rater agreement by item for quantitative studies 6

Table 4: Inter-rater agreement for overall scores of quantitative studies 7

Table 5: Inter-rater agreement by item for qualitative studies 8

Table 6: Inter-rater agreement for overall scores of qualitative studies 9

Table 7: Inter-rater agreement for paper inclusion/exclusion using a variety of cut-points for the overall scores in quantitative studies 10

Table 8: Inter-rater agreement for paper inclusion/exclusion using a variety of cut-points for the overall scores in quantitative studies 10

Discussion 11

References 12

Appendix A

Manual for Quality Scoring of Quantitative Studies 14

Appendix B

Manual for Quality Scoring of Qualitative Studies 20

Introduction

Systematic literature reviews, which have become increasingly common since the early 1990s, evolved in response to the shift towards evidence-based practice in medicine.[1,2] Systematic review methodology has largely focused on locating, evaluating and synthesizing information generated by randomized controlled trials (RCTs).[1] While RCTs likely provide more reliable information than other sources regarding the differential effectiveness of alternative forms of health care; systematic reviews of other types of evidence can facilitate decision-making in areas where RCTs have not been performed or are not appropriate.[3] In some research areas, limiting systematic reviews to the appraisal of RCTs may yield little or no information, yet there could be a great deal of other evidence to assess.[1]

> *... systematic reviews of other types of evidence can facilitate decision-making in areas where randomized controlled trials have not been performed or are not appropriate.*

We have recently undertaken a systematic review of the literature addressing the social, ethical and legal implications of genetic technologies used in cancer risk assessment. The review was limited to technologies that assist in the evaluation of an individual's genetic predisposition to developing cancer. Examples included tests for germline mutations in the adenomatous polyposis coli (APC) gene which is implicated in the dominant inheritance of familial adenomatous polyposis and the development of colorectal cancer[4] and in the breast cancer-associated genes BRCA1 and BRCA2 which is associated with hereditary breast and ovarian cancers.[5] Our search strategy, designed by a multi-disciplinary team, was developed with the goal of ensuring that a range of issues and literature was considered. Our search yielded a broad array of documents from both the peer-reviewed as well as the "gray" literature, ranging from primary reports of qualitative and quantitative research to narrative editorials and commentaries.

Our search of the published literature yielded 5,474 original records for initial review. Two reviewers independently screened the available titles and abstracts of these records and applied initial exclusion criteria. The reviewers were in agreement for 5,403/5,474 (98.7%) of the records. Discrepancies were resolved through discussion. For records where relevance could not be determined from the title and an abstract was not available, the document was retrieved for further review. Following this initial screen, documents consisting only of abstracts (n=87), review articles (n=43) and documents clearly not relevant to the topic at hand (n=4,649) were excluded. A total of 695 documents were selected for

retrieval. Of these, six could not be attained as the citations were invalid, and another 195 were excluded after further review (3 abstracts only, 24 review articles, 5 duplicate publications and 163 papers not relevant to the topic). Of the remaining 494 documents, 281 were narrative, non-research reports including editorials, commentaries, position statements, etc., 192 were reports of primary quantitative research and 21 were reports of primary qualitative research. This review is ongoing, and will be completed in early 2004.

To assess the quality of the primary research reports, we had originally proposed to use the checklist developed by the British Sociological Association Medical Sociology Group.[6] This checklist was designed specifically for use with qualitative studies and as a result did not easily lend itself to the evaluation of quantitative research. Our review, furthermore, differs from a number of published systematic reviews in that a single research question was not defined a priori. Rather, our review was designed to identify multiple important social, ethical and legal issues associated with cancer risk assessment technologies. We intentionally did not focus on a single issue, for example the effectiveness of a particular medical intervention, nor did we constrain the review to studies of a given design such as randomized controlled trials. The studies selected for retrieval thus covered a range of research topics and employed a number of designs.

... our review was designed to identify multiple important social, ethical and legal issues...

It has been suggested that hierarchical ordering of study designs (for example see Sackett)[7] can be used in systematic reviews to define a minimum quality threshold for study inclusion,[3,8] however, this was unsuitable for our review given the broad-based nature of the studies examined. Specifically, study designs were often expected to vary according to the issues addressed by the research questions. Our goal was to select, within topic areas, studies of sufficient quality for inclusion in the review. “Quality” was defined in terms of the internal validity of the studies, or the extent to which the design, conduct and analyses minimized errors and biases.[9] The need for standard, reproducible criteria to critically appraise the quality of the various studies was apparent.

Appraising the quality of evidence is an important, yet difficult task, complicated by the consideration of disparate evidence. Quality checklists for assessing RCTs abound,[2,10] yet it is acknowledged that even within this single study design the reliability, validity, feasibility and utility of the various tools are either unmeasured or quite variable.[2] To the best of our knowledge standard criteria for simultaneously assessing the quality of diverse study designs do not currently exist. Individual checklists have been adapted for use with other study designs such as Cho et al’s instrument for assessing the quality of observational and experimental but not randomized

drug studies[11] or alternate forms of research communications such as Timmer et al's quality scoring tool for abstracts.[12] Other more general tools are available, but have limited operational utility as the quality assessment criteria are largely focused on the quality of reporting, or specify items to use when abstracting data in a standard fashion from research reports, for example the evaluation tools for quantitative and qualitative studies developed by Health Care Practice Research and Development Unit.[13,14] The Cochrane Collaboration Non-Randomised Studies Methods Group is currently developing guidelines for the review of non-randomized studies, but the draft chapter on quality assessment is still pending.[15]

Methods

Given the lack of a standard, empirically grounded quality assessment tool suitable for use with a variety of study designs, we developed and implemented two scoring systems to evaluate the quality of the studies potentially eligible for inclusion in our review: one for quantitative research reports, and one for qualitative research reports. Our scoring systems draw upon existing published tools, relying particularly upon the instruments developed by Cho et al[11] and Timmer et al[12] for quantitative studies, and the guidelines suggested by Mays and Pope[16] and Popay et al[17] for qualitative studies. Our pragmatic systematic review tool "QualSyst" incorporates these two scoring systems.

Evaluating the quality of qualitative research, in particular, is a matter of considerable debate. Some maintain that qualitative research is a distinct paradigm defined by a commitment to relativism or anti-realism, and should not be subject to quality evaluation. Rejecting the idea that a single "reality" or "truth" exists independent of the research process,[16] supporters of this viewpoint maintain that people construct their own realities in different ways at different times and places, and the impossibility of a context-free reality precludes categorizing some versions of reality as "trustworthy."[18] Others contend that all research involves subjective perception, but that an underlying reality does exist and can be studied.[16] Supporters of this viewpoint argue that the same quality criteria (generally based on validity and reliability) should be applied to qualitative and quantitative research.[19] Finally, others argue that some quality criteria may be applied equally to the evaluation of both quantitative and qualitative research while other criteria may have to be modified to account for the particular features of qualitative research.[16,17]

While this conceptual debate is important, we nonetheless faced the practical challenge of simultaneously evaluating the quality of both

Table 1. Checklist for assessing the quality of quantitative studies

	Criteria	YES (2)	PARTIAL (1)	NO (0)	N/A
1	Question / objective sufficiently described?				
2	Study design evident and appropriate?				
3	Method of subject/comparison group selection or source of information/input variables described and appropriate?				
4	Subject (and comparison group, if applicable) characteristics sufficiently described?				
5	If interventional and random allocation was possible, was it described?				
6	If interventional and blinding of investigators was possible, was it reported?				
7	If interventional and blinding of subjects was possible, was it reported?				
8	Outcome and (if applicable) exposure measure(s) well defined and robust to measurement / misclassification bias? means of assessment reported?				
9	Sample size appropriate?				
10	Analytic methods described/justified and appropriate?				
11	Some estimate of variance is reported for the main results?				
12	Controlled for confounding?				
13	Results reported in sufficient detail?				
14	Conclusions supported by the results?				

types of research. We determined that it was not feasible to develop a single, operational scoring system capturing the central notions of "quality" described in the literature as relevant to both qualitative and quantitative reports. We, therefore, developed two separate systems. Rather than developing explicit definitions for the two types of research, our distinction between the two was practical. Studies employing quantitative methods were appraised using the system for quantitative studies, while studies identified by the researchers as qualitative or employing qualitative methods such as focus groups, semi-structured interviews, etc.[20] were appraised using the system for qualitative studies.

Table 2. Checklist for assessing the quality of qualitative studies

	Criteria	YES (2)	PARTIAL (1)	NO (0)
1	Question / objective sufficiently described?			
2	Study design evident and appropriate?			
3	Context for the study clear?			
4	Connection to a theoretical framework / wider body of knowledge?			
5	Sampling strategy described, relevant and justified?			
6	Data collection methods clearly described and systematic?			
7	Data analysis clearly described and systematic?			
8	Use of verification procedure(s) to establish credibility?			
9	Conclusions supported by the results?			
10	Reflexivity of the account?			

The original checklists and scoring manuals were developed following a review of various quality assessment documents and discussion by the authors of the elements considered central to internal study validity. Ten quantitative and ten qualitative studies were then randomly selected and independently scored by two reviewers. For the quantitative studies, 14 items (Table 1) were scored depending on the degree to which the specific criteria were met ("yes" = 2, "partial" = 1, "no" = 0). Items not applicable to a particular study design were marked "n/a" and were excluded from the calculation of the summary score. A summary score was calculated for each paper by summing the total score obtained across relevant items and dividing by the total possible score (i.e.: 28 – (number of "n/a" x 2)). Scores for the qualitative studies were calculated in a similar fashion, based on the scoring of ten items (Table 2). Assigning "n/a" was not permitted for any of the items, and the summary score for each paper was calculated by summing the total score obtained across the ten items and dividing by 20 (the total possible score).

Table 3. Inter-rater agreement by item for quantitative studies

Checklist Item	Observed Agreement for Each Checklist Item (%)	
	First Sample (n = 10)	Second Sample (n = 11)
1	60.0	100.0
2	90.0	90.9
3	90.0	100.0
4	70.0	100.0
5	n/a	n/a
6	n/a	n/a
7	n/a	n/a
8	60.0	81.8
9	60.0	72.7
10	80.0	90.9
11	70.0	100.0
12	40.0	90.9
13	70.0	90.9
14	60.0	90.9

Results

Evaluation of quantitative research

For the quantitative studies, inter-rater agreement in scoring (by item) ranged from 40% to 100% (Table 3). The overall scores (Table 4) assigned by the first reviewer ranged from 0.44 to 0.90 (mean: 0.76, standard deviation: 0.16). The overall scores assigned by the second reviewer ranged from 0.56 to 0.93 (mean: 0.80, standard deviation: 0.13). Both reviewers assigned the same overall score to two studies. For the remaining eight studies, discrepancies in the overall scores ranged from 0.02 to 0.12. Most discrepancies reflected differences of opinion on the applicability of certain items to specific study designs and on the assignment of "yes" versus "partial" to the fulfillment of specific criteria. Items where disagreement occurred were discussed and the checklists and accompanying manuals were revised substantially.

Table 4. Inter-rater agreement for overall scores of quantitative studies

Research Paper	Overall Score			
	First Sample		Second Sample	
	Rater 1	Rater 2	Rater 1	Rater 2
1	.44	.56	.73	.73
2	.86	.90	.73	.77
3	.73	.73	.59	.73
4	.89	.89	.55	.55
5	.89	.93	.50	.45
6	.68	.80	.82	.86
7	.55	.60	.68	.73
8	.90	.85	.90	.80
9	.82	.80	.73	.77
10	.82	.90	.50	.60
11	–	–	.40	.40

Given the substantial changes that were made to the quantitative checklist and scoring manual, a second sample of quantitative studies (5% or 11 studies) was randomly selected and scored independently by the same two reviewers. Inter-rater agreement for this sample is shown in Tables 3 and 4. Compared with the first sample, by-item agreement improved considerably, ranging from 73% to 100%. The overall scores assigned by the first reviewer ranged from 0.40 to 0.90 (mean: 0.65, standard deviation: 0.15). The overall scores assigned by the second reviewer ranged from 0.40 to 0.86 (mean: 0.67, standard deviation: 0.15). Both reviewers assigned the same overall score to 3 (27%) papers. Discrepancies for the remaining eight papers ranged from 0.04 to 0.14. This time, most discrepancies reflected differences in the assignment of "yes" versus "partial" to specific items. There was no disagreement on the applicability of specific items to different study designs. At this point, the scoring system for the quantitative studies was deemed suitably reproducible (see Appendix A for the final quality scoring manual). Evaluation of the remaining studies included in the systematic review, including re-evaluation of the original sample of ten studies, is currently underway.

Table 5. Inter-rater agreement by item for qualitative studies

Checklist Item	Observed Agreement (%) (n=10)
1	80.0
2	100.0
3	90.0
4	60.0
5	80.0
6	70.0
7	80.0
8	80.0
9	60.0
10	80.0

Evaluation of qualitative research

For the sample of ten qualitative studies, inter-rater agreement (by item) ranged from 60% to 100% (Table 5). As with the second sample of quantitative studies, most discrepancies reflected differences in the assignment of "yes" versus "partial" to specific items. The overall scores (Table 6) assigned by the first reviewer ranged from 0.55 to 0.90 (mean: 0.77, standard deviation: 0.11). The overall scores assigned by the second reviewer ranged from 0.65 to 0.85 (mean: 0.76, standard deviation: 0.06). Both reviewers assigned the same score to one study, and for all but one of the remaining nine studies, discrepancies in the overall scores ranged from 0.05 to 0.10. At this point, following minor revisions to the wording of a few checklist items, the scoring system for the qualitative studies was deemed suitably reproducible (see Appendix B for the final quality scoring manual). Evaluation of the remaining studies in the systematic review is underway.

The quality scores will be used to define a minimum threshold for inclusion of studies in the systematic review. This threshold will be determined by considering both the distribution of the quality scores and the time and resource constraints of the project. Whether the cut-point selected for article inclusion is relatively conservative (e.g., 75%) or relatively liberal (e.g., 55%), comparing the overall scores assigned by the two reviewers shows the scoring systems for both quantitative and qualitative studies to be relatively robust across a variety of plausible cut-points (Tables 7 & 8).

Table 6. Inter-rater agreement for overall scores of qualitative studies

Research Paper	Overall Score	
	Rater 1	Rater 2
1	.55	.65
2	.75	.80
3	.75	.80
4	.85	.85
5	.75	.80
6	.90	.75
7	.85	.75
8	.75	.70
9	.65	.70
10	.85	.80

In addition to informing the selection of a minimum threshold, the quality scores will also provide quantitative information on the relative quality of studies selected for inclusion in the review. Detailed assessment of differences in the scores within study designs,and across research paradigms, should prove useful when synthesizing information and exploring the heterogeneity of study results.

While the QualSyst tool has proven useful in the course of our work, it has limitations. First, the use of summary scores to identify high quality studies can, in itself, introduce bias into a systematic review. For example, Juni et al applied 25 different quality scales to 17 clinical trials comparing two types of heparin for the prevention of postoperative thrombosis and found that the type of scale used influenced the results of meta-analyses.[21] Our checklists are admittedly subjective and reflect our perceptions of the key components of study quality, defined in terms of internal study validity. Given the absence of standard operational definitions of internal validity in the literature and the absence of a "gold standard" to compare our tool with, we cannot be certain that our tool accurately measures what it is supposed to measure. However, our tool may facilitate discussion of this issue, and ultimately development of superior tools.

Second, our assessment of inter-rater reliability was limited. Practical time and resource constraints in the context of this project prevented us from reviewing a larger number of studies and estimating standard statistical

Table 7. Inter-rater agreement for paper inclusion/exclusion using a variety of cut-points for the overall scores in quantitative studies

Possible Cut-Point for Exclusion of Paper	Agree to Include # (%)	Agree to Exclude # (%)	Disagreement # (%)
< .55	8 (73)	2 (18)	1 (9)
< .60	6 (55)	3 (27)	2 (18)
< .65	6 (55)	4 (36)	1 (9)
< .70	5 (45)	4 (36)	2 (18)
< .75	2 (18)	7 (64)	2 (18)

Table 8. Inter-rater agreement for paper inclusion/exclusion using a variety of cut-points for the overall scores in qualitative studies

Possible Cut-Point for Exclusion of Paper	Agree to Include # (%)	Agree to Exclude # (%)	Disagreement # (%)
< .55	10 (100)	0 (0)	0 (0)
< .60	9 (90)	0 (0)	1 (10)
< .65	9 (90)	0 (0)	1 (10)
< .70	8 (80)	1 (10)	1 (10)
< .75	7 (70)	2 (20)	1 (10)

measures of agreement, for instance Kappa coefficients and related confidence intervals. Further, assessment of inter-rater agreement by a range of reviewers from both the quantitative and qualitative research arenas who were not involved in the development of the tool would increase our confidence in reliability. Funding is currently being sought to pursue this work.

Discussion

We have implemented a scoring system that provides a systematic, reproducible and quantitative means of simultaneously assessing the quality of research encompassing a broad range of study designs. QualSyst will ensure that studies ultimately selected to inform our systematic review meet a minimum quality standard. In the context of each identified research theme, it will also assist in the exploration of variation across studies and in the synthesis and interpretation of the research findings. We believe that our approach may prove useful to other investigators faced with the challenge of evaluating disparate sources of evidence, and hopefully will encourage further research in systematic review methodology.

QualSyst will ensure that studies ultimately selected to inform our systematic review meet a minimum quality standard.

References

1. Hawker S, Payne S, Kerr C, Hardey M, Powell J. Appraising the evidence: reviewing disparate data systematically. *Qual Health Res.*12:1284-99.
2. Lohr KN, Carey TS. Assessing "best evidence": issues in grading the quality of studies for systematic reviews. Jt Comm J Qual Improv. 1999;25:470-9.
3. Clarke, M., Oxman, A. D. (editors). *Cochrane Reviewer's Handbook* 4.2.0 [Updated March 2003]. In: The Cochrane Library, Issue 2, 2003. Oxford, Update Software (updated quarterly)
4. Fearnhead NS, Wilding JL, Bodmer WF. Genetics of colorectal cancer: hereditary aspects and overview of colorectal tumorigenesis. *Br Med Bull.* 2002;64:27-43.
5. Radice P. Mutations of BRCA genes in hereditary breast and ovarian cancer. *J Exp Clin Cancer Res.* 2002;21(3 Suppl):9-12.
6. Blaxter M. Criteria for evaluation of qualitative research. *Medical Sociology News*. 1996;22:68-71.
7. Sackett DL. Guidelines. In: Sackett DL, Strauss S, Richardson W, Rosenberg W, Haynes R. *Evidence-based medicine : how to practice and teach EBM*, 2nd ed. Edinburgh: Churchill Livingstone, 2000.
8. Khan, K. S., ter Riet, G., Glanville, J., Sowden, A. J., and Kleijnen, J. (editors) Undertaking systematic reviews of research on effectiveness: CRD's guidance for those carrying out or commissioning reviews. *CRD Report Number* 4, 2nd ed. 2001. York: NHS Centre for Reviews and Dissemination.
9. Hennekens CL, Buring JE. *Epidemiology in Medicine*, 1st ed. Boston: Little, Brown & Co., 1987.
10. Moher D, Jadad AR, Nichol G, Penman M, Tugwell P, Walsh S. Assessing the quality of randomized controlled trials: an annotated bibliography of scales and checklists. *Control Clin Trials.* 1995;16:62-73.
11. Cho MK, Bero LA. Instruments for assessing the quality of drug studies published in the medical literature. *JAMA.* 1994;272:101-4.
12. Timmer A, Sutherland LR, Hilsden RJ. Development and evaluation of a quality score for abstracts. *BMC Med Res Methodol.* 2003;3:2.
13. Health Care Practice Research and Development Unit – The University of Salford. Evaluation tools for quantitative studies. Available from: http://www.fhsc.salford.ac.uk/hcprdu/tools/quantitative.htm (accessed 2003 Sept 30)

14. Health Care Practice Research and Development Unit – The University of Salford. Evaluation tools for qualitative studies. Available from: http://www.fhsc.salford.ac.uk/hcprdu/tools/qualitative.htm. (accessed 2003 Sept 30)

15. Cochrane Collaboration Non-Randomised Studies Methodology Group. Draft chapters for the guidelines on non-randomised studies in Cochrane reviews. Available from: http://www.cochrance.dk/nrsmg/guidelines.htm. (accessed 2003 Sept 30)

16. Mays N, Pope C. Quality in qualitative health research. In: Mays N, Pope C. *Qualitative research in health care*, 2nd ed. London: BMJ Books, 2000:89-101.

17. Popay R, Rogers A, Williams G. Rationale and standards for the systematic review of qualitative literature in health services research. *Qual Health Res.* 1998;8:341-51.

18. Smith J. The problem of criteria for judging interpretive inquiry. *Educational Evaluation and Policy Analysis.* 1984;6:379-91.

19. Murphy E, Dingwall R, Greatbatch D, Parker S, Watson P. Qualitative research methods in health technology assessment: a review of the literature. *Health Technol Assess*. 1998;2(16):iii-ix, 1-274.

20. Pope C, Mays N. Qualitative methods in qualitative health research. In: Mays N, Pope C. *Qualitative research in health care*, 2nd ed. London: BMJ Books, 2000:1-10.

21. Juni P, Witschi A, Bloch R, Egger M. The hazards of scoring the quality of clinical trials for meta-analysis. *JAMA.* 1999;282:1054-60.

Appendix A: Manual for Quality Scoring of Quantitative Studies

Definitions and Instructions for Quality Assessment Scoring

How to calculate the summary score

- **Total sum** = (number of "yes" * 2) + (number of "partials" * 1)
- **Total possible sum** = 28 – (number of "N/A" * 2)
- **Summary score:** total sum / total possible sum

Quality assessment

1. *Question or objective sufficiently described?*

   Yes: Is easily identified in the introductory section (or first paragraph of methods section). Specifies (where applicable, depending on study design) *all* of the following: purpose, subjects/target population, and the *specific* intervention(s) /association(s)/descriptive parameter(s) under investigation. A study purpose that only becomes apparent after studying other parts of the paper is *not* considered sufficiently described.

   Partial: Vaguely/incompletely reported (e.g. "describe the effect of" or "examine the role of" or "assess opinion on many issues" or "explore the general attitudes"...); *or* some information has to be gathered from parts of the paper other than the introduction/background/objective section.

   No: Question or objective is not reported, or is incomprehensible.

   N/A: Should not be checked for this question.

2. *Design evident and appropriate to answer study question?*

   (If the study question is not given, infer from the conclusions).

   Yes: Design is easily identified and is appropriate to address the study question / objective.

   Partial: Design and /or study question not clearly identified, but gross inappropriateness is not evident; *or* design is easily identified but only partially addresses the study question.

   No: Design used does not answer study question (e.g., a comparison group is required to answer the study question, but none was used); *or* design cannot be identified.

   N/A: Should not be checked for this question.

3. *Method of subject selection (and comparison group selection, if applicable) or source of information/input variables (e.g., for decision analysis) is described and appropriate.*

Yes: Described and appropriate. Selection strategy *designed* (i.e., consider sampling frame and strategy) to obtain an unbiased sample of the relevant target population or the entire target population of interest (e.g., consecutive patients for clinical trials, population-based random sample for case-control studies or surveys). Where applicable, inclusion/exclusion criteria are described and defined (e.g., "cancer" -- ICD code or equivalent should be provided). *Studies of volunteers:* methods and setting of recruitment reported. *Surveys:* sampling frame/ strategy clearly described and appropriate.

Partial: Selection methods (and inclusion/exclusion criteria, where applicable) are not completely described, but no obvious inappropriateness. Or selection strategy is not ideal (i.e., likely introduced bias) but did not likely seriously distort the results (e.g., telephone survey sampled from listed phone numbers only; hospital based case-control study identified all cases admitted during the study period, but recruited controls admitted during the day/evening only). Any study describing participants only as "volunteers" or "healthy volunteers". *Surveys:* target population mentioned but sampling strategy unclear.

No: No information provided. *Or* obviously inappropriate selection procedures (e.g., inappropriate comparison group if intervention in women is compared to intervention in men). *Or* presence of selection bias which likely seriously distorted the results (e.g., obvious selection on "exposure" in a case-control study).

N/A: Descriptive case series/reports.

4. *Subject (and comparison group, if applicable) characteristics or input variables/information (e.g., for decision analyses) sufficiently described?*

Yes: Sufficient relevant baseline/demographic information clearly characterizing the participants is provided (or reference to previously published baseline data is provided). Where applicable, reproducible criteria used to describe/categorize the participants are clearly defined (e.g., ever-smokers, depression scores, systolic blood pressure > 140). If "healthy volunteers" are used, age and sex must be reported (at minimum). *Decision analyses:* baseline estimates for input variables are clearly specified.

Partial: Poorly defined criteria (e.g. "hypertension", "healthy volunteers", "smoking"). *Or* incomplete relevant baseline / demographic information (e.g., information on likely confounders not reported). *Decision analyses:* incomplete reporting of baseline estimates for input variables.

No: No baseline / demographic information provided.
Decision analyses: baseline estimates of input variables not given.

N/A: Should not be checked for this question.

5. *If random allocation to treatment group was possible, is it described?*

Yes: True randomization done - requires a description of the method used (e.g., use of random numbers).

Partial: Randomization mentioned, but method is not (i.e. it may have been possible that randomization was not true).

No: Random allocation not mentioned although it would have been feasible and appropriate (and was possibly done).

N/A: Observational analytic studies. Uncontrolled experimental studies. Surveys. Descriptive case series / reports. Decision analyses.

6. *If interventional and blinding of investigators to intervention was possible, is it reported?*

Yes: Blinding reported.

Partial: Blinding reported but it is not clear who was blinded.

No: Blinding would have been possible (and was possibly done) but is not reported.

N/A: Observational analytic studies. Uncontrolled experimental studies. Surveys. Descriptive case series / reports. Decision analyses.

7. *If interventional and blinding of subjects to intervention was possible, is it reported?*

Yes: Blinding reported.

Partial: Blinding reported but it is not clear who was blinded.

No: Blinding would have been possible (and was possibly done) but is not reported.

N/A: Observational studies. Uncontrolled experimental studies. Surveys. Descriptive case series / reports.

8. *Outcome and (if applicable) exposure measure(s) well defined and robust to measurement / misclassification bias? Means of assessment reported?*

Yes: Defined (or reference to complete definitions is provided) and measured according to reproducible, "objective" criteria (e.g., death, test completion – yes/no, clinical scores). Little or minimal potential for measurement / misclassification errors. *Surveys:* clear description (or reference to clear description) of questionnaire/interview content and response options. *Decision analyses:* sources of uncertainty are defined for all input variables.

Partial: Definition of measures leaves room for subjectivity, *or* not sure (i.e., not reported in detail, but probably acceptable). *Or* precise definition(s) are missing, but no evidence or problems in the paper that would lead one to assume major problems. *Or* instrument/mode of assessment(s) not reported. Or misclassification errors may have occurred, but they did not likely seriously distort the results (e.g., slight difficulty with recall of long-ago events; exposure is measured only at baseline in a long cohort study). *Surveys:* description of

questionnaire/interview content incomplete; response options unclear. *Decision analyses:* sources of uncertainty are defined only for some input variables.

No: Measures not defined, or are inconsistent throughout the paper. *Or* measures employ only ill-defined, subjective assessments, e.g. "anxiety" or "pain." *Or* obvious misclassification errors/measurement bias likely seriously distorted the results (e.g., a prospective cohort relies on self-reported outcomes among the "unexposed" but requires clinical assessment of the "exposed"). *Surveys:* no description of questionnaire/interview content or response options. *Decision analyses:* sources of uncertainty are not defined for input variables.

N/A: Descriptive case series / reports.

9. *Sample size appropriate?*

Yes: Seems reasonable with respect to the outcome under study and the study design. When statistically significant results are achieved for major outcomes, appropriate sample size can usually be assumed, unless large standard errors (SE > ½ effect size) and/or problems with multiple testing are evident. *Decision analyses:* size of modeled cohort / number of iterations specified and justified.

Partial: Insufficient data to assess sample size (e.g., sample seems "small" and there is no mention of power/sample size/effect size of interest and/or variance estimates aren't provided). *Or* some statistically significant results with standard errors > ½ effect size (i.e., imprecise results). *Or* some statistically significant results in the absence of variance estimates. *Decision analyses:* incomplete description or justification of size of modeled cohort / number of iterations.

No: Obviously inadequate (e.g., statistically non-significant results and standard errors > ½ effect size; or standard deviations > _ of effect size; or statistically non-significant results with no variance estimates and obviously inadequate sample size). *Decision analyses:* size of modeled cohort / number of iterations not specified.

N/A: Most surveys (except surveys comparing responses between groups or change over time). Descriptive case series / reports.

10. *Analysis described and appropriate?*

Yes: Analytic methods are described (e.g. "chi square"/ "t-tests"/"Kaplan-Meier with log rank tests", etc.) and appropriate.

Partial: Analytic methods are not reported and have to be guessed at, but are probably appropriate. *Or* minor flaws or some tests appropriate, some not (e.g., parametric tests used, but unsure whether appropriate; control group exists but is not used for statistical analysis). *Or* multiple testing problems not addressed.

No: Analysis methods not described and cannot be determined. *Or* obviously inappropriate analysis methods (e.g., chi-square tests for continuous data, SE given where normality is highly unlikely, etc.). *Or* a study with a descriptive goal / objective is over-analyzed.

N/A: Descriptive case series / reports.

11. *Some estimate of variance (e.g., confidence intervals, standard errors) is reported for the main results/outcomes (i.e., those directly addressing the study question/ objective upon which the conclusions are based)?*

Yes: Appropriate variances estimate(s) is/are provided (e.g., range, distribution, confidence intervals, etc.). *Decision analyses:* sensitivity analysis includes all variables in the model.

Partial: Undefined "+/-" expressions. *Or* no specific data given, but insufficient power acknowledged as a problem. *Or* variance estimates not provided for all main results/outcomes. *Or* inappropriate variance estimates (e.g., a study examining change over time provides a variance around the parameter of interest at "time 1" or "time 2", but does not provide an estimate of the variance around the difference). *Decision analyses:* sensitivity analysis is limited, including only some variables in the model.

No: No information regarding uncertainty of the estimates. *Decision analyses:* No sensitivity analysis.

N/A: Descriptive case series / reports. Descriptive surveys collecting information using open-ended questions.

12. *Controlled for confounding?*

Yes: Randomized study, with comparability of baseline characteristics reported (or non-comparability controlled for in the analysis). *Or* appropriate control at the design or analysis stage (e.g., matching, subgroup analysis, multivariate models, etc). *Decision analyses:* dependencies between variables fully accounted for (e.g., joint variables are considered).

Partial: Incomplete control of confounding. *Or* control of confounding reportedly done but not completely described. *Or* randomized study without report of comparability of baseline characteristics. *Or* confounding not considered, but not likely to have seriously distorted the results. *Decision analyses:* incomplete consideration of dependencies between variables.

No: Confounding not considered, and may have seriously distorted the results. *Decision analyses:* dependencies between variables not considered.

N/A: Cross-sectional surveys of a single group (i.e., surveys examining change over time or surveys comparing different groups should address the potential for confounding). Descriptive studies. Studies explicitly stating the analysis is strictly descriptive/exploratory in nature.

13. *Results reported in sufficient detail?*

Yes: Results include major outcomes and all mentioned secondary outcomes.

Partial: Quantitative results reported only for some outcomes. *Or* difficult to assess as study question/objective not fully described (and is not made clear in the methods section), but results seem appropriate.

No: Quantitative results are reported for a subsample only, or "n" changes continually across the denominator (e.g., reported proportions do not account for the entire study sample, but are reported only for those with complete data

-- i.e., the category of "unknown" is not used where needed). *Or* results for some major or mentioned secondary outcomes are only qualitatively reported when quantitative reporting would have been possible (e.g., results include vague comments such as "more likely" without quantitative report of actual numbers).

N/A: Should not be checked for this question.

14. *Do the results support the conclusions?*

Yes: All the conclusions are supported by the data (even if analysis was inappropriate). Conclusions are based on all results relevant to the study question, negative as well as positive ones (e.g., they aren't based on the sole significant finding while ignoring the negative results). Part of the conclusions may expand beyond the results, if made *in addition to* rather than instead of those strictly supported by data, and if including indicators of their interpretative nature (e.g., "suggesting," "possibly").

Partial: Some of the major conclusions are supported by the data, some are not. *Or* speculative interpretations are not indicated as such. *Or* low (or unreported) response rates call into question the validity of generalizing the results to the target population of interest (i.e., the population defined by the sampling frame/strategy).

No: None or a very small minority of the major conclusions are supported by the data. *Or* negative findings clearly due to low power are reported as definitive evidence against the alternate hypothesis. *Or* conclusions are missing. *Or* extremely low response rates invalidate generalizing the results to the target population of interest (i.e., the population defined by the sampling frame/strategy).

N/A: Should not be checked for this question.

Appendix B: Manual for Quality Scoring of Qualitative Studies

Definitions and Instructions for Quality Assessment Scoring

How to calculate the summary score

- **Total sum** = (number of "yes" * 2) + (number of "partials" * 1)
- **Total possible sum** = 20
- **Summary score:** total sum / total possible sum

Quality assessment

1. *Question / objective clearly described?*

   Yes: Research question or objective is clear by the end of the research process (if not at the outset).

   Partial: Research question or objective is vaguely/incompletely reported.

   No: Question or objective is not reported, or is incomprehensible.

2. *Design evident and appropriate to answer study question?*

   (If the study question is not clearly identified, infer appropriateness from results/conclusions.)

   Yes: Design is easily identified and is appropriate to address the study question.

   Partial: Design is not clearly identified, but gross inappropriateness is not evident; *or* design is easily identified but a different method would have been more appropriate.

   No: Design used is not appropriate to the study question (e.g. a causal hypothesis is tested using qualitative methods); *or* design cannot be identified.

3. *Context for the study is clear?*

   Yes: The context/setting is adequately described, permitting the reader to relate the findings to other settings.

   Partial: The context/setting is partially described.

   No: The context/setting is not described.

4. *Connection to a theoretical framework / wider body of knowledge?*

   Yes: The theoretical framework/wider body of knowledge informing the study and the methods used is sufficiently described and justified.

Partial: The theoretical framework/wider body of knowledge is not well described or justified; link to the study methods is not clear.

No: Theoretical framework/wider body of knowledge is not discussed.

5. *Sampling strategy described, relevant and justified?*

Yes: The sampling strategy is clearly described and justified. The sample includes the full range of relevant, possible cases/settings (i.e., more than simple convenience sampling), permitting conceptual (rather than statistical) generalizations.

Partial: The sampling strategy is not completely described, or is not fully justified. Or the sample does not include the full range of relevant, possible cases/settings (i.e., includes a convenience sample only).

No: Sampling strategy is not described.

6. *Data collection methods clearly described and systematic?*

Yes: The data collection procedures are systematic, and clearly described, permitting an "audit trail" such that the procedures could be replicated.

Partial: Data collection procedures are not clearly described; difficult to determine if systematic or replicable.

No: Data collection procedures are not described.

7. *Data analysis clearly described, complete and systematic?*

Yes: Systematic analytic methods are clearly described, permitting an "audit trail" such that the procedures could be replicated. The iteration between the data and the explanations for the data (i.e., the theory) is clear – it is apparent how early, simple classifications evolved into more sophisticated coding structures which then evolved into clearly defined concepts/explanations for the data). Sufficient data is provided to allow the reader to judge whether the interpretation offered is adequately supported by the data.

Partial: Analytic methods are not fully described. *Or* the iterative link between data and theory is not clear.

No: The analytic methods are not described. *Or* it is not apparent that a link to theory informs the analysis.

8. *Use of verification procedure(s) to establish credibility of the study?*

Yes: One or more verification procedures were used to help establish credibility/ trustworthiness of the study (e.g., prolonged engagement in the field, triangulation, peer review or debriefing, negative case analysis, member checks, external audits/inter-rater reliability, "batch" analysis).

No: Verification procedure(s) not evident.

9. *Conclusions supported by the results?*

   Yes: Sufficient original evidence supports the conclusions. A link to theory informs any claims of generalizability.

   Partial: The conclusions are only partly supported by the data. *Or* claims of generalizability are not supported.

   No: The conclusions are not supported by the data. *Or* conclusions are absent.

10. *Reflexivity of the account?*

    Yes: The researcher explicitly assessed the likely impact of their own personal characteristics (such as age, sex and professional status) and the methods used on the data obtained.

    Partial: Possible sources of influence on the data obtained were mentioned, but the likely impact of the influence or influences was not discussed.

    No: There is no evidence of reflexivity in the study report.
